# Hepatic Involvement across the Metabolic Syndrome Spectrum: Non-Invasive Assessment and Risk Prediction Using Machine Learning

**DOI:** 10.3390/jcm12175657

**Published:** 2023-08-30

**Authors:** Adelaida Solomon, Călin Remus Cipăian, Mihai Octavian Negrea, Adrian Boicean, Romeo Mihaila, Corina Beca, Mirela Livia Popa, Sebastian Mihai Grama, Minodora Teodoru, Bogdan Neamtu

**Affiliations:** 1 Faculty of Medicine, “Lucian Blaga” University, 550024 Sibiu, Romania; solomonadelaida@gmail.com (A.S.); adrian.boicean@ulbsibiu.ro (A.B.); romeo.mihaila@ulbsibiu.ro (R.M.); liviamirelapopa@yahoo.com (M.L.P.); sebastian.grama@ulbsibiu.ro (S.M.G.); minodora.teodoru@ulbsibiu.ro (M.T.); bogdan.neamtu@ulbsibiu.ro (B.N.); 2 County Clinical Emergency Hospital of Sibiu, 2–4 Corneliu Coposu Str., 550245 Sibiu, Romania; corina.beca@gmail.com; 3 Department of Clinical Research, Pediatric Clinical Hospital Sibiu, 550166 Sibiu, Romania

**Keywords:** metabolic syndrome, non-alcoholic fatty liver disease, metabolic-associated fatty liver disease, non-invasive tests, transient elastography, liver stiffness measurement, cluster analysis, decision tree algorithms

## Abstract

Metabolic-dysfunction-associated steatotic liver disease (MASLD) and metabolic syndrome (MetS) are inextricably linked conditions, both of which are experiencing an upward trend in prevalence, thereby exerting a substantial clinical and economic burden. The presence of MetS should prompt the search for metabolic-associated liver disease. Liver fibrosis is the main predictor of liver-related morbidity and mortality. Non-invasive tests (NIT) such as the Fibrosis-4 index (FIB4), aspartate aminotransferase-to-platelet ratio index (APRI), aspartate aminotransferase-to-alanine aminotransferase ratio (AAR), hepatic steatosis index (HIS), transient elastography (TE), and combined scores (AGILE3+, AGILE4) facilitate the detection of liver fibrosis or steatosis. Our study enrolled 217 patients with suspected MASLD, 109 of whom were diagnosed with MetS. We implemented clinical and biological evaluations complemented by transient elastography (TE) to discern the most robust predictors for liver disease manifestation patterns. Patients with MetS had significantly higher values of FIB4, APRI, HSI, liver stiffness, and steatosis parameters measured by TE, as well as AGILE3+ and AGILE4 scores. Machine-learning algorithms enhanced our evaluation. A two-step cluster algorithm yielded three clusters with reliable model quality. Cluster 1 contained patients without significant fibrosis or steatosis, while clusters 2 and 3 showed a higher prevalence of significant liver fibrosis or at least moderate steatosis as measured by TE. A decision tree algorithm identified age, BMI, liver enzyme levels, and metabolic syndrome characteristics as significant factors in predicting cluster membership with an overall accuracy of 89.4%. Combining NITs improves the accuracy of detecting patterns of liver involvement in patients with suspected MASLD.

## 1. Introduction

MASLD is a subcategory of a condition previously termed non-alcoholic fatty liver disease (NAFLD). NAFLD was observed to impact approximately a quarter of the world’s population, and its prevalence is increasing in parallel with the escalating rates of obesity and type 2 diabetes [1,2,3]. In addition, a noteworthy association has been established between NAFLD and cerebrovascular disease [4]. NAFLD was predicted to become the first indication for a liver transplant by 2030 [5]. In its previous definition, this disease encompassed a spectrum ranging from simple steatosis to steatohepatitis, liver fibrosis, and potentially cirrhosis and hepatocellular carcinoma [6]. The underlying mechanisms are complex and multifactorial, with the disbalance between excessive nutrient delivery through the portal vein and an insufficient complementary oxygen supply increase via the hepatic artery possibly playing a pivotal role in leading to hepatic injury [7]. The conglomeration of such dysmetabolic mechanisms within patients with liver steatosis is well documented, with metabolic syndrome (MetS) being a significant risk factor for its development and progression. Metabolic syndrome is a constellation of conditions that include central obesity, dyslipidemia (low high-density cholesterol, high triglycerides), hyperglycemia, and hypertension [8]. The definitions of MetS differ slightly according to the criteria used, but three out of five components are generally required to diagnose the disease. The number and severity of the metabolic abnormalities correlate with the severity of liver disease and the risk of advanced fibrosis, the main predictors of liver-related morbidity and mortality [9,10,11]. The classic of for NAFLD entails evidence of hepatic steatosis in the absence of excessive alcohol consumption and secondary causes of liver steatosis. The name NAFLD fails to capture the metabolic component of the disease, which is crucial for disease progression [12]. To reflect the growing recognition of the metabolic dysfunction that underlies this condition and to emphasize the importance of addressing the metabolic risk factors, a new terminology has been proposed—metabolic-dysfunction-associated fatty liver disease (MAFLD) [13]. In June 2023, the American Association for the Study of Liver Disease (AASLD) and the European Association for the Study of the Liver (EASL), in collaboration with the Asociación Latinoamericana para el Estudio del Hígado (ALEH), published the Delphi consensus on the new fatty liver disease nomenclature [14]. “Steatotic liver disease” (SLD) is the new general overarching term that encompasses the most common causes of steatosis: Metabolic-dysfunction-associated steatotic liver disease (MASLD), alcohol-related liver disease (ALD), and an overlap of the two termed MetALD (MASLD and increased alcohol intake). The subcategories of SLD also include specific aetiologies (monogenic diseases, drug-induced liver injury, celiac disease, Wilson disease, viral hepatitis), and if there are no identifiable cardiometabolic risk factors or specific causes of steatosis, this is termed cryptogenic SLD. According to the proposed definition, the presence of at least one cardiometabolic risk factor, in addition to hepatic steatosis (diagnosed histologically or by imaging), is required for diagnosing MASLD. The cardiometabolic risk factors overlap with the criteria for the diagnosis of metabolic syndrome, with the addition of a higher-than-normal body mass index to the criteria. The aim is to identify patients with insulin resistance as the primary cause of hepatic steatosis [15].

In the absence of identifiable cardiometabolic factors or specific aetiologies for SLD (thus fitting the cryptogenic SLD category), clinicians can assign this particular setting the term “possible MASLD” if they suspect metabolic dysfunction. Such patients could develop cardiometabolic risk factors and benefit from regular assessments.

Conceptually, individuals who previously fell under the NAFLD definition are now entirely encompassed within the categories of MASLD and possible MASLD, as demonstrated by a comprehensive analysis conducted on the European cohort of the LITMUS consortium. The results revealed that 98% of the patients previously categorized as having NAFLD now meet the criteria for MASLD [16].

The proposed terminology represents an improvement over the previous “non-alcoholic” label. It establishes a metabolic foundation for this liver disease, which has long been acknowledged as “the hepatic manifestation of the metabolic syndrome” [17]. These new terms aim to provide a positive and non-stigmatizing description of the condition, moving away from a diagnosis of exclusion.

The presence of liver steatosis is required for the diagnosis of MASLD. This is frequently achieved through imaging. The magnetic resonance imaging techniques proton density fat fraction (PDFF) and magnetic resonance spectroscopy (MRS) are highly accurate for quantifying liver steatosis. While MRS is considered one of the most accurate non-invasive methods for quantifying liver steatosis, its limited availability and high cost reduce its accessibility [18]. Meanwhile, efforts have been made to improve the time efficiency of magnetic resonance imaging proton density fat fraction (MRI-PDFF)-based liver steatosis quantification by automating the segmentation process involved [19]. Nevertheless, conventional ultrasound remains the first-line tool. Still, it is limited in patients with obesity; it has high inter-operator variability; and it cannot quantify steatosis or fibrosis [20]. Standard ultrasound has a low sensitivity to detect hepatic steatosis, but it is often the initial investigation in practice. The hepatic steatosis index (HSI) is a validated screening tool recommended by the European Association for the Study of the Liver (EASL) [21]. It employs simple variables such as AST, ALT, diabetes status, and gender to estimate the likelihood of hepatic steatosis. The endorsement is attributed to its high accuracy and straightforward calculation using readily available medical record data [22]. Liver steatosis can also be quantified by the controlled attenuation parameter obtained using transient elastography [23].

The main predictor of liver disease progression is fibrosis. Currently, liver biopsy is the gold standard for diagnosing SLD. It is, however, an invasive procedure with potential complications and limitations, which lead to significant difficulties in its implementation on a large scale. The European Association for the Study of the Liver (EASL) strongly recommends the use of non-invasive techniques such as liver stiffness measurement (LSM) via transient elastography (TE) and serological scores (FIB4, APRI) to assess and stratify the risk of liver-related outcomes in individuals with MASLD. More than one method should be used to improve liver fibrosis estimation accuracy. Repeated measurements of non-invasive tests can help further refine the risk stratification for liver-related events in patients with MASLD and non-alcoholic steatohepatitis (NASH), currently termed metabolic-dysfunction-associated steatohepatitis (MASH). Although there is a lack of conclusive evidence regarding the optimal timing for subsequent LSM assessments, previous recommendations suggested repeating NITs every three years for patients in the early phases of NAFLD in its classic definition and annually for those with more advanced stages. However, this recommendation is considered weak due to the limited supporting evidence [21].

The Delphi consensus remains in line with previous case definitions for steatohepatitis and disease stages. The diagnosis of MASLD/MASH with advanced fibrosis/cirrhosis, even when steatosis may not be present, will be based on existing agreed-upon criteria for NASH cirrhosis. This also applies to patients with significant fibrosis, who may not have steatosis but are categorized under the overarching term of SLD, reflecting the mechanism of injury [24].

In this study, we aimed to investigate the patterns of non-invasive markers that quantify the extension of liver steatosis and the degree of fibrosis in patients with suspected MASLD while accounting for the presence of MetS components. Using a CART decision tree, we stratified patients to identify those at risk of liver disease progression who would benefit from TE and subsequent closer monitoring. The CART decision tree is valuable due to its adaptability for various data types and distributions, robustness against anomalies, and effective handling of missing data through automated alternate divisions. The automated mechanism identifies optimal partition points (threshold values) for predictive variables, demonstrating adaptability in practical applications. The validity of using decision trees, such as the Classification and Regression Tree (CART) algorithm, for predicting outcomes based on a combination of individual traits is supported in the review article by Venkatasubramaniam, A. et al. [25]. The review uses the “Box Lunch Study”, a trial on a sample of 233 adults investigating how portion size availability affects caloric intake and weight gain, to demonstrate the successful application of decision trees in predicting outcomes based on a combination of individual characteristics [26].

This approach aligns with the methodology of our own study, where we used the CART algorithm to classify patients with liver steatosis and fibrosis based on clinical indicators and metabolic syndrome components. Combining clinical and biological data in machine-learning algorithms improves prediction accuracy and highlights an advanced approach in alignment with precision medicine principles.

The findings of this study emphasize the overlap of the pathogenesis of MetS and MASLD and support the adoption of the new terminology and the use of simple blood tests, BMI, and the MetS criteria to identify those at risk of advanced liver disease.

## 2. Materials and Methods

### 2.1. Study Protocol

We conducted an analytical, prospective observational study from January 2021 to January 2023 in the Medical Department of Sibiu Clinical County Hospital. Each patient signed an informed consent form to participate, and the research was conducted with the approval of the Ethics Committee of the Sibiu Clinical County Hospital. The patients were referred to our tertiary hospital for further evaluation after a suspicion of MASLD was raised in different centers due to ultrasonographic evidence of liver steatosis. The ultrasonographic presence of liver steatosis was then confirmed at our center. Data collection started before the new nomenclature for MASLD was established and, consequently, employed inclusion criteria that defined the entity of NAFLD. Our inclusion/exclusion approach was conceptually similar to the study conducted by Eletreby et al. [27].

Patients with possible alcohol-related liver disease (ALD) or MetALD were excluded from our study based on self-reported lack of significant alcohol consumption (defined as <20 g/day for women and <30 g/day for men) [14,28].

In addition, patients with any disease that could explain liver involvement within the SLD spectrum were excluded. This was achieved via thorough evaluation and exclusion of viral and autoimmune hepatitis, hemochromatosis, Wilson disease, celiac disease, heart failure with liver congestion, and medication-induced liver injury in all the included patients.

Consequently, the included subjects were diagnosed with NAFLD as previously defined and encompassed patients who, according to the new nomenclature for fatty liver disease, fell into either the category of MASLD or possible MASLD/cryptogenic SLD. The latter applied to patients with a normal BMI where no evidence of metabolic syndrome components was identified.

Each patient underwent clinical and paraclinical examinations (including TE) during the same visit. We recorded past medical history, medication history, age, gender, height, weight, body mass index (BMI), waist circumference, blood pressure, heart rate, full blood count, liver function tests, lipid profile, and fasting plasma glucose.

Weight categorization adhered to the cut-offs established by the Center for Disease Control and Prevention for BMI [29,30]: 18.5–24.99 kg/m² for normal weight, 25–29.99 kg/m²; 25–29.99 kg/m² for overweight, 30–34.99 kg/m² for class 1 obesity, 35–39.99 kg/m² for class 2 obesity, and ≥40 kg/m for class 3 obesity.

The chosen diagnostic criteria for metabolic syndrome were guided by the standards set by the European Association for the Study of the Liver (EASL), European Association for the Study of Diabetes (EASD), and European Association for the Study of Obesity (EASO) in the 2016 Clinical Practice Guidelines for the Management of Non-Alcoholic Fatty Liver Disease: Waist circumference ≥ 94/80 cm for men/women, arterial pressure ≥ 130/85 mm Hg or treated for hypertension, fasting glucose ≥ 100 mg/dL or treated for type 2 diabetes mellitus (T2DM), serum triglycerides > 150 mg/dL, and HDL cholesterol < 40/50 mg/dL for men/women [31]. The number of metabolic components present in each patient was recorded (0–5).

Vibration-controlled transient elastography (VCTE) measurements were obtained by an experienced operator using the Fibroscan device. We adhered to the quality criteria for transient elastography from Fibroscan: All the patients were required to fast for at least 3 h before the examination; a minimum of 10 measurements were obtained; and the interquartile range was confined to under 30%. The probe (M or XL) was chosen according to the patient’s morphology and the device’s tool for probe selection. Measurements were conducted on the right hepatic lobe through the intercostal space while the patient was lying in a dorsal decubitus position with the right arm maximally abducted. Liver stiffness measurement (LSM), indicating fibrosis, and the controlled attenuation parameter (CAP), indicating steatosis, were simultaneously obtained. The final LSM, expressed in kilopascal (kPa) units, and the final CAP, expressed in decibels per meter (dB/m), were determined based on the median value of the ten acquisitions. The success rate was calculated as the ratio of successful measurements to the total number of measurements. Only procedures meeting the criteria of at least ten valid measurements, a success rate of at least 60%, and an interquartile range (IQR)/median value of LSM ≤ 0.3 were considered reliable, while examinations failing to meet these criteria were excluded from the analysis. Steatosis was graded into the following four categories using the thresholds provided by the meta-analysis by Karlas T. et al.: no steatosis CAP < 234 dB/m, mild steatosis CAP 234−268 dB/m, moderate steatosis CAP 269–300 dB/m, and severe steatosis CAP ≥ 301 dB/m [32].

Similarly, fibrosis measured using VCTE categorized patients into five groups: no fibrosis LSM < 6.5 kPa (F0), incipient fibrosis 6.5–7.1 kPa (F1), moderate fibrosis 7.2–9.5 kPa (F2), advanced fibrosis 9.6–14.4 kPa (F3), and cirrhosis ≥ 14.5 kPa (F4). These cut-offs are based on a meta-analysis of studies that juxtaposed the outcomes of transient elastography and liver biopsy [33].

The serological and clinical data obtained were used for calculating the hepatic steatosis index (HSI), AST-to-platelet ratio index (APRI), FIB4, and AST/ALT ratio (AAR).

HSI values below 30 indicate that NAFLD can be ruled out with a sensitivity of 93.1%, and at values above 36, HSI detected NAFLD with a specificity of 92.4% [22].

APRI can predict outcomes in patients with NAFLD based on its previous definition [34,35]. According to the National Institute for Clinical Excellence (NICE), a value of 0.5 can be used to rule in/out significant fibrosis [36].

Regarding the FIB4 score, a cut-off value <1.3 rules out significant fibrosis [37,38,39].

AAR was used in predictive models for patients with NAFLD according to its previous definition, with a cut-off value <0.8 for ruling out fibrosis [40].

In addition, we used the data obtained via TE to compute the Agile3+ and Agile4 scores. These scores have values between 0 and 1 and can be viewed as probabilities for patients with suspected MASLD to have advanced fibrosis (Agile3+) or cirrhosis (Agile4) [41]. In addition, Agile 3+ has recently been shown to predict liver-related events in patients with suspected MASLD [42].

The mathematical equations for the scores mentioned above are provided in Supplementary Materials Table S1.

### 2.2. Statistical Analysis

The statistical analysis of the data was conducted using the IBM SPSS Statistics 21 software suite. Continuous variables were characterized using mean and median values, standard deviation, 95% confidence interval, minimum, maximum, and interquartile range. The frequency distributions were calculated for categorical variables. The Shapiro–Wilk test was employed to assess the normality of the quantitative variables. For continuous variables that adhered to a normal distribution, t-Student tests were employed for comparison purposes, while variables with a skewed distribution were analyzed using the Mann–Whitney U test. The chi-square test or Fisher exact test was applied to assess for the presence of significant relationships between categorical variables. Results were considered statistically significant if *p* < 0.05.

### 2.3. Machine-Learning Approach

We performed a two-step cluster analysis to delineate patient groups regarding the degree of liver damage defined via TE. Subsequently, we implemented a classification and regression tree (CART) to explore a set of discriminative rules to distinguish between the defined groups [43].

Two-step cluster analysis is a method that combines K-means and hierarchical cluster methods to group observations based on their shared traits. The characteristics selected in our approach were represented by the results obtained after performing TE. A two-stage process utilizing the Akaike Information Criterion (AIC) determined the number of clusters. We considered an average silhouette of cohesion separation above 0.5 as an indicator of the model’s good quality.

Decision trees are supervised machine-learning methods that categorize data, thereby uncovering concealed patterns behind user-defined outcomes. This yields a graphic decision model centered around the target variable (the cluster membership generated in the previous step). The model is built in reverse, starting from the top root and branching out until splits cease, linking all predictors to forecast the overall outcome. These branches occur based on conditions (internal nodes) applied to predictor variables, leading to further splits and decisions. The terminal point of a branch (“leaf” or child node) represents the final decision [43].

Criteria for tree growth may involve selecting the longest path length from the top root to a child node or choosing a minimum number of training inputs for each child node. CART is a binary splitting decision tree that employs the Gini index and entropy rule to partition data based on predictor variables and node purity, proceeding from parent to child node. The best solution that significantly increases node purity is chosen from all the feasible splitting paths. This process recurs until the stopping criteria are met, or no further reduction in node impurity is possible. The main goal is to identify the best split point (cut-off value) for a predictor variable, maximizing the splitting criteria based on the Gini index, the Twoing impurity measure for categorical variables, or LSD (least squares deviation) impurity measure for continuous variables. The algorithm then determines the best node split by selecting the predictor that maximizes the splitting criterion, resulting in the highest reduction in node impurity. The process repeats for each “child” node until no further improvement can be achieved or predefined stopping rules are satisfied. Typically, the minimum improvement threshold is user-specified and is often set at 0.0001. The CART decision tree exhibits versatility in handling different data types and distributions, robustness against outliers, and effective handling of missing values through surrogate splits via its fully automated mechanism.

The CART models were computed in pruning mode, considering variables that were either part of the definition of the metabolic syndrome or part of the simple scores calculated to identify liver fibrosis or steatosis. We first analyzed the correlations between these scores and metabolic syndrome using standard statistical methods. Successive exhaustive additions or removals of component variables were employed to find the optimal model. To grow the decision tree model, we selected 10 maximum growth levels, with 5 as the minimum number of cases for parent nodes and 3 for child nodes. Regarding the Gini impurity measure, a minimum change in improvement of 0.0001 was set to circumvent overfitting (maximum difference in risk in standard errors: 0).

We fed the following continuous variables to the algorithm: age, BMI, ALT, AST, platelet count, and three of the five dichotomous definitory characteristics of metabolic syndrome: hypertriglyceridemia or treatment for dyslipidemia, low HDL cholesterol or treatment for dyslipidemia, and fasting glucose ≥100 mg/dL or treatment for type 2 diabetes mellitus.

After the tree has grown to its full depth until stopping criteria are met, pruning trims the tree down (removing the nodes that provide less additional information) to the smallest subtree with an acceptable risk value. Pruned CART models perform cross-validation using cost-complexity approaches for trimming to minimize the average of the mean square prediction errors and increase the stability of the model.

The CART algorithm identifies subgroups based on combinations of traits without attempting to provide a causal explanation for the defined rule set. Unlike linear regression, the CART algorithm is a nonlinear method. Its automatic pruning provides robustness against multicollinearity, making it less susceptible to confounders in our specific dataset and context.

**Figure 1:**
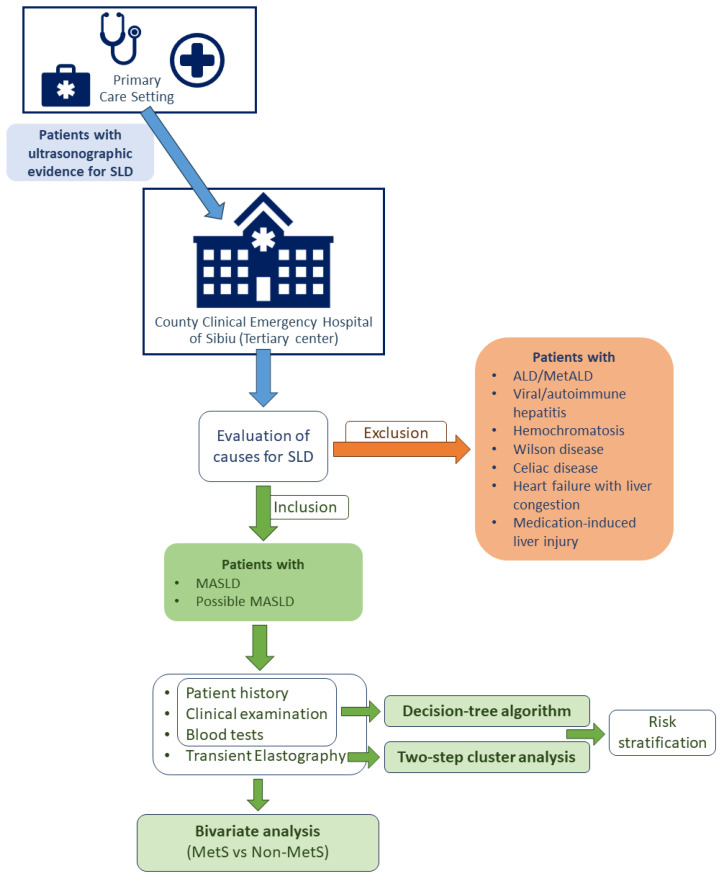
Study workflow.

Figure 1 provides a graphic representation of the study’s workflow.

## 3. Results

A total of 217 patients with suspected MASLD were included in the study. In total, 126 were female and 91 were male. The ages of the participants ranged between 19 and 85. Of the patients, 109 fulfilled the criteria for metabolic syndrome, and 108 did not. All the included patients were Caucasian.

### 3.1. Description of the Study Population

#### 3.1.1. General Characteristics and Measured Parameters

The variables analyzed were age, body mass index (BMI), AST (aspartate aminotransferase), ALT (alanine aminotransferase), platelets, CAP (controlled attenuation parameter), E (liver stiffness measurement), APRI (aspartate aminotransferase-to-platelet ratio index), FIB 4 (Fibrosis-4 index), AAR (AST/ALT ratio), Agile 3+ score, Agile 4 score, and HSI (hepatic steatosis index). Statistically significant differences between genders are presented in Table S2 in the Supplementary Materials. Women had higher BMI, platelet count, AAR, and HSI and lower ALT and APRI when compared to the men in our study. Age, AST, CAP, E, FIB4, Agile 3+, and Agile 4 showed no statistically significant differences between genders.

The gender distribution was similar between patients with and without MetS as well as within each of the five defining criteria of MetS. The mean age was 53.10 for the group without MetS and 62.78 for the group with MetS. The distribution of the studied parameters between patients with and without MetS is presented in detail in Table S3 in the Supplementary Materials, with corresponding significance levels for the statistical tests employed to investigate the differences between groups. Except for AAR, all of the variables showed a statistically significant association with metabolic syndrome.

#### 3.1.2. Distribution of Weight Categories

Of the 217 patients, 55 had a normal weight (50 without MetS and only 5 from the MetS group). The data suggest a higher prevalence of overweight and obesity (class I, II, and III) among individuals with MetS compared to those without MetS, with statistically significant differences observed across the different BMI categories (*p* < 0.05), as presented in Table 1.

### 3.2. Study Population According to the New Fatty Liver Disease Nomenclature

According to the new nomenclature for fatty liver disease, 91.71% of the included patients met the criteria for the diagnosis of MASLD. A total of 18 patients (8.29%) had no discernible cardiometabolic component associated with steatotic liver disease and were consequently classified as cryptogenic SLD.

The most frequent metabolic abnormality found in our study population was high fasting glucose or treated T2DM (65.44% of the group without MetS and 88.99% of the group with MetS), followed by hypertension (29.63% for the group without MetS and 86.24% of those with MetS) and high waist circumference (20.37% of those without MetS and 74.31% of the MetS group) (Table 2).

### 3.3. Analysis of Liver Steatosis and Fibrosis

#### 3.3.1. Steatosis Measured Using Transient Elastography

Steatosis was measured using TE, with Fibroscan, using the controlled attenuation parameter. The results are presented in Table 3.

Only 8.25% of the patients with MetS had no steatosis, as determined via TE. Mild steatosis (S1) was found in 11.92%, moderate steatosis (S2) in 27.52%, and severe steatosis (S3) in 52.29%. Higher proportions of individuals with metabolic syndrome exhibit higher degrees of steatosis than those without.

#### 3.3.2. Fibrosis Measured via Transient Elastography

Most of the patients without MetS (88.89%) had no fibrosis (LSM < 6.5 kPa). Significant fibrosis (F2, F3) was found in 36.7% and cirrhosis in 10.09% among those with MetS and only in 3.71% and 2.78%, respectively, for the patients without MetS. Details are presented in Table 4.

#### 3.3.3. Machine-Learning Algorithms for Risk Stratification

We used a two-step cluster algorithm using Akaike’s information criterion based on the CAP (dB/m) and E (kPa) values, allowing for automatic cluster number determination. The result was a model defining three clusters with an average silhouette of cohesion separation of 0.6, indicating a good model quality. Table 5 provides an overview of the model and the obtained clusters’ characteristics.

Patients in cluster 3 were more prone to significant liver fibrosis and steatosis, while patients in cluster 2 had an increased chance of having at least moderate steatosis compared to cluster 1. A detailed comparison between clusters is presented in Tables S4 and S5 in the Supplementary Materials. We then combined clusters 2 and 3. The resulting data are presented in Table 6.

The merging of clusters 2 and 3 yielded a subpopulation of patients who more frequently had either significant liver fibrosis or at least moderate liver steatosis, as quantified via VCTE. Of the 18 patients with no cardiometabolic criteria, 17 (94.44%) were grouped within cluster 1.

We performed a CART decision tree algorithm to find the relevant predictors for patients to fall into the category represented by the merged cluster.

**Figure 2:**
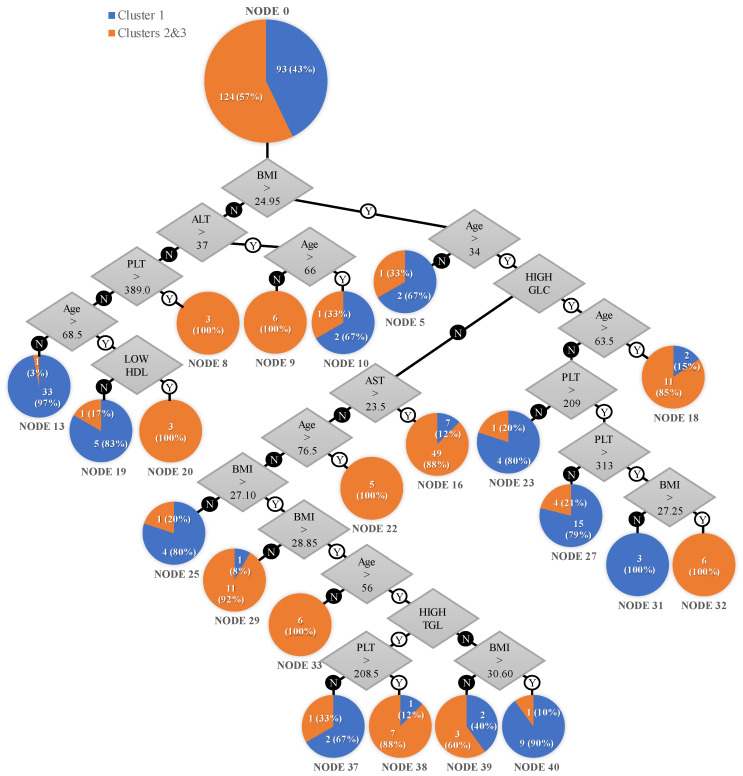
Decision tree predicting cluster membership.

Figure 2 presents the rules within the algorithm as well as the final resulting nodes. The detailed CART algorithm can be found in the Supplementary Materials Figure S1.

The resulting model could predict membership in cluster 1 with 86% accuracy and membership in the merged clusters 2 and 3 with 91.9% accuracy (an overall accuracy of 89.4%).

Our CART algorithm led to decision paths indicating distinct risk categories for the presence of liver disease determined by the results of VCTE (clusters 2 and 3, merged). Among the twenty-one terminal nodes, twenty identified risks either below 33% or exceeding 85%, except for a single node (37) indicating a 60% risk. This outcome suggests the algorithm’s effective capacity to stratify individuals at risk of liver disease. The following populations comprised the risk groups defined by the decision tree algorithm:

High-risk group:

- A total of 12 patients with a normal BMI (<24.95 kg/m²), 6 aged under 66, with an ALT > 37 U/L, 3 with an AST < 37 U/L and platelet count > 389 × 10^9^/L, and 3 aged over 68.5, with an ALT < 37 U/L and low HDL cholesterol, all 12 (100%) belonging to the hepatic involvement cluster.
- A total of 17 patients with a high BMI (>24.95 kg/m²), 5 aged over 76.5, 6 aged 34–63.5 with diabetes mellitus or impaired fasting glucose, BMI > 27.25, and platelet count > 313 × 10^9^/L, and 6 aged 34–56 with a BMI > 28.85 kg/m², all 17 (100%) belonging to the hepatic involvement cluster.
- A total of 56 patients with a BMI > 24.95 kg/m², aged > 34, with an AST > 23.5 U/L, of which 49 (88%) were in the hepatic involvement cluster and 7 (12%) were in cluster 1.
- A total of 12 patients with a BMI ranged 27.1–28.85 kg/m², aged 34–76.5, with an AST < 23.5 U/L, of which 11 (92%) were in the hepatic involvement cluster and 1 (8%) was in cluster 1.
- A total of 13 patients with a BMI > 24.95 kg/m², aged > 63.5, with diabetes mellitus or impaired fasting glucose of whom 11 (85%) were in the hepatic involvement cluster and 2 (15%) were in cluster 1.
- A total of 8 patients with a BMI > 28.85 kg/m², aged 56–76.5, with high triglycerides and platelet count > 208.5 × 10^9^/L, of whom 7 (88%) were in the hepatic involvement cluster and 1 (12%) was in cluster 1.

Medium-risk group:

- A total of 5 patients with a BMI ranged 28.85–30.6 kg/m², aged over 56, with an AST < 23.5 U/L and normal fasting glucose, of whom 2 (40%) belonged to cluster 1 and 3 (60%) were in the hepatic involvement cluster.

Low-risk group:

- A total of 3 patients with a BMI > 24.95 kg/m², aged under 34, of whom 1 (33%) was in the hepatic involvement cluster and 2 (67%) were in cluster 1.
- A total of 3 patients with a BMI < 24.95 kg/m², aged over 66, with an ALT > 37 U/L, of whom 1 (33%) was in the hepatic involvement cluster and 2 (67%) were in cluster 1.
- A total of 34 patients with a BMI < 24.95 kg/m², aged under 68.5, with an ALT < 37 U/L and platelet count < 389 × 10^9^/L, of which 1 (3%) was in the hepatic involvement cluster and 33 (97%) were in cluster 1.
- A total of 6 patients with a BMI < 24.95 kg/m², aged under 68.5, with an ALT < 37 U/L and platelet count < 389 × 10^9^/L, with normal HDL cholesterol, of whom 1 (17%) was in the hepatic involvement cluster and 5 (83%) were in cluster 1.
- A total of 5 patients with a BMI > 24.95 kg/m², aged 34–63.5, with diabetes mellitus or impaired fasting glucose, platelet count < 209 × 10^9^/L, of whom 1 (20%) was in the hepatic involvement cluster and 4 (80%) were in cluster 1.
- A total of 9 patients, aged 34–63.5 with diabetes mellitus or impaired fasting glucose, platelet count of 209–313 × 10^9^/L, of whom 4 (21%) were in the hepatic involvement cluster and 15 (79%) were in cluster 1.
- A total of 10 patients with a BMI > 30.60 kg/m², aged 56–76.5, with normal triglycerides and AST < 23.5 U/L, of whom 1 (10%) was in the hepatic involvement cluster and 9 (90%) were in cluster 1.
- A total of 5 patients with a BMI ranged 24.95–27.1 kg/m², aged 34–76.5, AST < 23.5 U/L, of whom 1 (20%) was in the hepatic involvement cluster and 4 (80%) were in cluster 1.
- A total of 3 patients with BMI > 28.85 kg/m², aged 56–76.5, with high triglycerides and platelet count < 208.5 × 10^9^/L, of whom 1 (33%) was in the hepatic involvement cluster and 2 (67%) were in cluster 1.
- A total of 3 patients with a BMI ranged 24.95–27.25 kg/m², aged 34–63.5, with diabetes mellitus or impaired fasting glucose, platelet count > 313 × 10^9^/L, of whom 0 (0%) were in the hepatic involvement cluster and 3 (100%) were in cluster 1.

**Figure 3:**
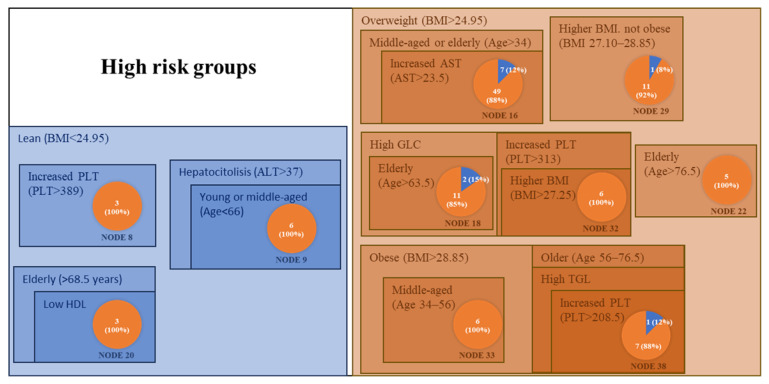
High risk subpopulations identified by the CART algorithm.

A BMI above 24.95 was the first rule to stratify patients. Patients with a normal BMI under the age of 66 with an ALT value above 37 UI/L or above the age of 68.5 with low HDL or a platelet count above 389 10×/L were assigned to clusters 2 and 3. Patients with a BMI above 24.95 with diabetes mellitus or impaired fasting glucose above the age of 63.5 were assigned to clusters 2 and 3 in 85% of cases. In contrast, patients without diabetes or impaired fasting glucose were assigned to clusters 2 and 3 if they were above the age of 76.5 or in a proportion of 92% if they had a BMI between 27.10 and 28.85. Patients without diabetes mellitus or impaired fasting glucose with a BMI above 28.85 under the age of 56 were assigned to clusters 2 and 3, while those above the age of 56 were assigned to clusters 2 and 3 in a proportion of 88% if they had increased triglycerides and platelet count. Figure 3 illustrates the various subpopulations within the high-risk group based on their defining characteristics, as determined by the CART algorithm.

## 4. Discussion

This study compared liver steatosis and fibrosis in a group of 109 people with MetS with a group of 108 people without MetS and found significantly higher rates of occurrence in those with MetS. This is consistent with the data reported in the literature. Multiple studies have shown that the presence of obesity, T2DM, and dyslipidemia increases the risk of NAFLD according to its previous definition [44,45,46,47].

### 4.1. Characteristics of Patients with MetS

#### 4.1.1. Demographics

The gender distribution reported for the metabolic syndrome differs with the population studied but tends to be equal. This study’s higher proportion of female participants may be attributed to several factors. Some studies have shown that the risk of developing MetS increases with age, particularly in women. This can be attributed to hormonal changes associated with menopause, including decreased estrogen levels, which can contribute to weight gain, redistribution of body fat, and metabolic changes [48]. The same mechanism could also explain the differences we found between genders concerning BMI in our study.

As expected, age was higher in the MetS group—the mean age was 62.78 vs. 53.10 in those without MetS [49].

The most frequent component of the MetS group was FG/T2DM. This is different from other studies that reported waist circumference and might be due to the growing prevalence of T2DM and the lowering of the cut-off for abnormal FG from 110 to 100 mg/dL, as proposed by the National Cholesterol Education Program Adult Treatment Panel III in their revised criteria from 2005 [50].

It could also show the important role of insulin resistance in the pathogenesis of liver steatosis and metabolic dysfunction [8].

#### 4.1.2. Liver Involvement

AST and ALT values differed between groups, with higher values in the MetS patients. Although normal liver function tests cannot reliably exclude steatohepatitis, elevated liver enzymes are a marker of inflammation and can indicate the presence of NASH. The AAR was not significantly different between the two groups. The utility of this ratio for quantifying fibrosis remains uncertain, as reported earlier in the literature [51].

Several studies have reported a considerable number of false-negative results when utilizing the FIB-4 score [52]. Its performance tends to be inadequate in individuals with obesity or advanced age [53]. Therefore, relying solely on the FIB-4 score, especially in patients with MASLD, is not advised.

TE improves the prediction of liver fibrosis when it is performed after other serum scores [54]. Using scores that combine clinical and serological characteristics with results obtained from transient elastography may enhance diagnostic accuracy [42]. The models we derived from machine-learning algorithms in our study could aid in establishing which patient profiles are most likely to be diagnosed with significant liver fibrosis or steatosis using TE, thus benefiting the most from this investigation concerning risk stratification.

The CART algorithm was employed to scrutinize patient data encompassing metabolic syndrome components, liver enzymes (AST, ALT), BMI, platelet count, age, HDL cholesterol, and triglycerides. The objective was to classify patients based on their propensity for liver fibrosis and steatosis. The algorithm effectively partitioned the patient cohort into two clusters: One characterized by reduced likelihood of liver fibrosis and steatosis (cluster 1), and another grouping patients with moderate to severe liver involvement (merged clusters 2 and 3). This method facilitated the identification of distinct patient subgroups with varying disease risks, thus aiding clinical stratification.

The decision tree algorithm identified an interesting finding: The first predictor selected was a BMI of 24.95 kg/m², which closely aligns with the cut-off for normal weight classification. This suggests that BMI plays a significant role in distinguishing different risk profiles within the study population. Utilizing a two-step cluster algorithm successfully yielded three distinct clusters with a reliable model quality. This highlights the effectiveness of this algorithm in stratifying patients based on their characteristics. Patients in cluster 3 were more prone to significant liver fibrosis and steatosis, while patients in cluster 2 had an increased chance of having at least moderate steatosis compared to those in cluster 1. For the grading of liver steatosis according to CAP, we used the data from the meta-analysis by Karlas T. et al. [32]. Interestingly, however, cluster 2, which contained patients with higher degrees of liver steatosis, all had values for this parameter of at least 276 db/m. The 2021 update of the EASL Clinical Practice Guidelines on non-invasive tests for the evaluation of liver disease severity and prognosis [21] cites two studies aiming to explore the value of CAP in quantifying liver fibrosis and suggests that cut-off values of 263 dB/m [55] and 274 dB/m [56] could detect liver steatosis of above 5% with sensitivities and positive predictive values of above 90%. A meta-analysis also cited in the EASL guidelines found a sensitivity of above 90% for the detection of any steatosis in patients with suspected MASLD for the cut-off value of 263 dB/m (95% CI 256–270) [57]. The guidelines conclude that, while there is no current consensus regarding the cut-off for CAP, values exceeding 275 dB/m are highly sensitive in predicting liver steatosis. The results we have obtained by implementing a two-step cluster analysis are in agreement with these values.

Age, BMI, liver enzyme levels, and metabolic syndrome characteristics were identified as significant factors for stratification. These factors contribute to the differentiation of patients within the clusters and help in understanding the underlying risk profiles.

With regard to the precise patient profiles unearthed through the application of the decision tree algorithm, normal BMI acted as a protective factor in most patients, except for young patients (<66 years) with higher ALT values (node 9) or older patients (>68.5 years) with low HDL (node 20) or a relatively higher platelet count (node 8). BMI showed an incremental predictive value in patients under the age of 76.5 (node 29).

While increased age is a recognized risk factor for suspected MASLD, and our findings align with this concept (node 22), there is a known heterogeneity concerning enzymatic and metabolic changes across age groups in patients developing SLD. Older patients exhibit a stronger association between glucose metabolism impairment or dyslipidemia and liver affliction compared to the predictive value of BMI [58]. The results from our decision tree are in agreement with this concept, as older patients with key defining elements of metabolic syndrome in our study, namely altered glucose metabolism (node 18), low HDL (node 20), or high triglycerides (node 40), showed an increased risk of more advanced MASLD profiles. In our model, an impaired glucose metabolism improved patient classification on a higher hierarchical level compared to parameters linked to lipid metabolism dysfunction. Similar findings have been described in metabolically unhealthy obese patients undergoing bariatric surgery, where parameters describing glucose metabolism more accurately predicted MASHM compared to those related to lipid metabolism [59].

Younger patients were more prone to advanced MASLD if they were obese (node 33), while older patients required further stratification according to metabolic risk factors and platelet count (node 40). Platelet count in and of itself is a controversial matter in suspected MASLD. Traditionally, NAFLD is associated with a low platelet count, as documented by several studies and extending even to the development of validated risk scores making use of this correlation [29,32,33,34,35,36,37,38,39]. One theory suggests that these findings are linked to reduced thrombopoietin circulating levels as a consequence of liver damage and subsequent inadequate platelet production in the bone [60]. MASLD, however, is associated with an important inflammatory response targeting the liver as part of the disease’s fundamental mechanisms [61,62,63]. The link between platelet count and inflammation [64] might explain the lack of consensus in the literature concerning how exactly platelet count predicts liver damage in MASLD, with some research pointing towards a positive correlation between the two [60,65]. Our findings suggest a similar relationship in certain groups (node 8, node 32, and node 40). Liver enzymes in our decision tree also aided in patient stratification, but again with varying profiles according to age and weight status (nodes 9, 10, and 16).

Model accuracy: The resulting model demonstrated an overall accuracy of 89.4% in predicting cluster membership, implying its robustness and effectiveness in classifying patients into the appropriate risk categories. Other studies advocate using a two-tier approach in primary care to select patients to refer for TE after calculating the FIB-4 score [66]. This strategy has been proven to be cost efficient, as it also minimizes unnecessary referrals. The study suggests that combining NITs improves the accuracy of detecting liver fibrosis. By integrating multiple non-invasive tests, healthcare providers can enhance their ability to identify and assess liver fibrosis without the need for invasive procedures.

### 4.2. Strengths and Limitations

#### 4.2.1. Liver Biopsy

One of this study’s limitations is that liver fibrosis was not assessed using the gold standard (liver biopsy). Hence, the results obtained by using NITs could not be verified. It is important to note that discrepancies between NITs and liver biopsy may arise from potential inaccuracies in the sampling and staging process associated with biopsy [67]. Another limitation is that patients were referred to the hospital for screening for MASLD due to increased liver echogenicity. Consequently, our design did not allow for random patient selection and may have led to overestimating the presence of liver steatosis and fibrosis in the general population with MetS.

#### 4.2.2. Sample Size

Several key factors in relation to how we conducted our study may warrant a discussion concerning the internal validity of our findings. These elements refer to the sample size we utilized as well as the influence of possible extraneous variables influencing our results.

Sample size may be a limitation, particularly in data processing using machine-learning [68]. Several studies, however, have successfully implemented similar algorithms and methodologies to our approach in order to discern between certain dichotomous outcomes in a similar field to our own research.

Lu et al., for example, conducted a prospective study using clinical parameters and genetic biomarkers in a cohort of 55 patients displaying advanced liver fibrosis who achieved a sustained virologic response following antiviral therapy for hepatitis C. The researchers used the CART algorithm to predict the occurrence of hepatocellular carcinoma in eight of the 55 patients [69].

Another study using CART analysis in a sample of 98 COVID-19 patients investigated the impact of liver biochemistry changes, previous liver disease, and liver elastography on clinical outcomes. The researchers used the hepatic steatosis index (HSI) and the CAP for steatosis quantification. AST and ALT were also used as variables to assess liver involvement. Their goal was to assess the prognostic significance of liver elastography, including steatosis measurement, in the context of COVID-19. The CART method was effective in detecting relevant variable interactions and identifying patient subgroups sharing similar clinical characteristics and prognoses [70].

#### 4.2.3. Confounding Factors

The presence of possible extraneous variables influencing the outcomes measured in our study has been documented and adjusted for where feasible and appropriate. A point-by-point description of each such scenario follows.

Simple scores characterizing fibrosis or steatosis are predominantly derived from logistic regression or statistical methods leveraging clinical or laboratory data [21]. They provide an indirect measure of steatosis/fibrosis. With regard to how they may be influenced by confounding factors, FIB-4 and ASAT/ALAT have low specificity in patients aged over 65 and suboptimal performance in those under 35 [71], while APRI is susceptible to the influence of platelet count abnormalities in hematological or other diseases. HSI does not take into account the severity of the disease. Moreover, scores using irreversible factors such as age, sex, and ethnicity are inherently less suitable for describing disease progression. To circumvent the shortcomings of the computed scores, we fed the CART algorithm with the base variables used to calculate them. While these variables were each susceptible to being swayed by confounding factors individually as well, their effects as detected within the CART algorithm are physiopathologically sound, as explained above with regard to AST, ALT, and platelet count, which may be influenced by a possible oscillatory inflammatory status characteristic of patients with MASLD/MASH. Further influences on the observed parameters pertain to the iatrogenic aspects surrounding the cardiometabolic criteria necessary for the diagnosis of MASLD. This is, however, accounted for within the definition of each criterion. To exemplify, while low HDL cholesterol or elevated triglycerides are standalone criteria, lipid-lowering treatments that may obscure them are integrated as variants for these criteria definitions. A similar situation can be described for impaired fasting glucose, the presence of diabetes mellitus, or treatment for diabetes [71].

External influences on the aforementioned variables and risk scores were minimized by stringent inclusion and exclusion criteria. One limitation of our study, however, was the fact that alcohol consumption was self-reported.

Conceptually addressing the influences on the individual components of each risk score facilitated the identification of inhomogeneous populations and the definition of appropriate risk groups with the help of the CART algorithm. In addition, the algorithm did not seek to provide a solution for dynamic long-term assessment or causal effect explanation of the patterns found, but rather to predict which particular populations would most probably have altered results due to VCTE testing, and, as such, would be warranted to undergo this examination.

As for the outcome parameters, the use of a two-step cluster algorithm allowed for delineation between patients without significantly altering parameters as measured using VCTE (cluster 1), patients with high CAP (cluster 2), and patients with high E(kPa) (cluster 3). With regard to the confounding factors that may influence these parameters, it should be noted that CAP failure is higher in obese patients. The use of an XL probe can mitigate this drawback and was implemented where deemed necessary in our study. In addition, CAP accuracy is lower in older patients or those with significant fibrosis [71]. In order to adjust for this latter aspect, we made the decision to merge cluster 2, which defined patients with significant liver steatosis via CAP, with cluster 3, which defined patients with significant fibrosis, to create a binary outcome that essentially divided patients with notable abnormal findings when subjected to VCTE. Further factors that can skew Fibroscan results include ascites, acute hepatitis, extrahepatic cholestasis, liver congestion, excessive alcohol intake, food intake, and operator experience. We accounted for the latter two aspects by applying the quality criteria for transient elastography via Fibroscan described in our methodology. In addition, in order to avoid the other possible situations described above that could alter our results, we thoroughly screened patients for the aforementioned pathologies, which consisted of applying the exclusion criteria of our study.

### 4.3. Financial Aspects

The Fibroscan device is fairly expensive and not readily available in primary or secondary care settings in Romania. Transient elastography is not on the list of services paid for by the National Health Insurance. It is mostly available in tertiary care centers, such as academic hospitals. Therefore, assessment using readily available tools, such as simple blood tests and biochemical scores, is warranted for the initial evaluation of patients with MetS and MASLD. Our decision tree algorithm could help distinguish between patients who would most likely have abnormal results when performing TE, thus potentially improving cost efficiency.

### 4.4. Future Directions

Future research should focus on establishing a unified definition of MetS, adopting the terminology of MASLD, and exploring the synergistic relationship between these two entities. Establishing a standardized definition will facilitate accurate diagnosis, consistent research findings, and effective management strategies. By maintaining the term and clinical definition of steatohepatitis, previous data from clinical trials and biomarker discovery studies related to NASH patients remain relevant and applicable to individuals classified as having MASLD or MASH under the new nomenclature. This continuity allows for the retention and validity of prior research without hindering its efficiency. The term MASLD better reflects the underlying metabolic dysfunction and encompasses a broader spectrum of liver-related conditions. Patients can grasp the condition better when it is linked to underlying cardiometabolic abnormalities related to insulin resistance and its association with their other health conditions, rather than being perceived as a diagnosis of exclusion. This approach also facilitates effective communication about the necessary therapeutic measures from both a liver-specific and holistic standpoint. Adopting this classification is expected to raise disease awareness. By aligning the diagnostic criteria for MASLD with well-recognized phenotypic traits in diabetes and cardiovascular medicine, healthcare providers can more readily identify individuals with this condition. This shift in terminology will enable a more comprehensive approach to understanding the disease pathogenesis, identifying at-risk individuals, and implementing appropriate interventions.

## 5. Conclusions

Linking MetS and liver steatosis together can significantly enhance awareness and improve the diagnostic and monitoring processes of liver disease. Recognizing the strong association between MetS and MASLD will prompt screening for liver-related complications in individuals with metabolic risk factors. It is expected that in the near future, non-invasive tests will be utilized to enhance disease grading, which can be incorporated into future updates on disease stage clarification.

Machine-learning techniques can effectively leverage basic patient data, including age, BMI, MetS components, AST, and ALT, to select individuals who should undergo further evaluation using transient elastography. Given the high prevalence of MASLD and the necessity to identify those at risk of advanced liver disease, this approach proves to be both cost efficient and crucial.

This integrated approach will facilitate the early detection of liver disease and the implementation of targeted management approaches that address both metabolic and liver-related aspects.

## Figures and Tables

**Table 1:** Distribution of BMI categories.

Weight Category	Total	Metabolic Syndrome		*p*-Value
		No	Yes	
Normal	55 (25.35%)	50 (46.30%)	5 (4.59%)	<0.01
Overweight	76 (35.02%)	38 (35.19%)	38 (34.86%)	
Obesity class I	52 (23.96%)	15 (13.89%)	37 (33.94%)	
Obesity class II	23 (10.60%)	4 (3.70%)	19 (17.43%)	
Obesity class III	11 (5.07%)	1 (0.93%)	10 (9.17%)	

**Table 2:** Prevalence of cardiometabolic criteria in the study population.

Cardiometabolic Criteria	Total	Metabolic Syndrome		*p*-Value
		No	Yes	
BMI ≥ 25 kg/m^2^	162 (74.65%)	58 (53.7%)	104 (95.41%)	*p* < 0.01
WC	103 (47.47%)	22 (20.37%)	81 (74.31%)	*p* < 0.01
BP	126 (58.06%)	32 (29.63%)	94 (86.24%)	*p* < 0.01
FG/T2DM	142 (65.44%)	45 (41.67%)	97 (88.99%)	*p* < 0.01
TGL	80 (36.87%)	19 (17.59%)	61 (55.96%)	*p* < 0.01
HDL	75 (34.56%)	9 (8.33%)	66 (60.55%)	*p* < 0.01
None	18 (8.29%)	18 (16.67%)	0 (0%)	*p* < 0.01

where WC is waist circumference ≥ 94/80 cm for men/women; BP is arterial pressure ≥ 130/85 mm Hg or treated for hypertension; FG/T2DM is fasting glucose ≥ 100 mg/dL or treated for T2DM; TGL is serum triglycerides > 150 mg/dL or treated for dyslipidemia; and HDL is HDL cholesterol <40/50 mg/dL for men/women or treated for dyslipidemia.

**Table 3:** Steatosis degrees in the study population.

Steatosis	Total	Metabolic Syndrome		*p*-Value
		No	Yes	
No steatosis	45 (20.74%)	36 (33.33%)	9 (8.25%)	<0.01
Mild steatosis	39 (17.97%)	26 (24.07%)	13 (11.92%)	
Moderate steatosis	50 (23.04%)	20 (18.52%)	30 (27.52%)	
Severe steatosis	83 (38.25%)	26 (24.07%)	57 (52.29%)	

**Table 4:** Fibrosis degrees in the study population.

Fibrosis	Total	Metabolic Syndrome		*p*-Value
		No	Yes	
No fibrosis	142 (65.40%)	96 (88.89%)	46 (42.20%)	<0.01
F1	17 (7.80%)	5 (4.62%)	12 (11.01%)	
F2	23 (10.60%)	3 (2.78%)	20 (18.35%)	
F3	21 (9.70%)	1 (0.93%)	20 (18.35%)	
F4	14 (6.5%)	3 (2.78%)	11 (10.09%)	

**Table 5:** Two-step cluster analysis overview.

Variable	Characteristic	Cluster 1	Cluster 2	Cluster 3
Count	-	93	103	21
E (kPa)	Mean	4.99	6.19	19.01
	StdDev	1.93	2.18	7.38
	IQR	2.1	3.2	10.1
	MIN	2.1	2.1	11.4
	MAX	12.9	11.3	38
	95%CI	4.61–5.37	5.77–6.62	15.66–22.37
	Predictor importance	1		
CAP (dB/m)	Mean	231.63	318.53	312.81
	StdDev	34.42	29.44	50.5
	IQR	52	40	70
	MIN	101	276	219
	MAX	276	400	390
	95%CI	224.55–238.72	312.78–324.29	289.82–335.79
	Predictor importance		0.91	
Liver fibrosis	No fibrosis	71 (76.3%)	67 (65%)	4 (19.0%)
	F1	10 (10.8%)	6 (5.8%)	1 (4.8%)
	F2	7 (7.5%)	14 (13.6%)	2 (9.5%)
	F3	4 (4.3%)	11 (10.7%)	6 (28.6%)
	F4	1 (1.1%)	5 (4.9%)	8 (38.1%)
Liver steatosis	No steatosis	38 (40.9%)	6 (5.8%)	1 (4.8%)
	Mild steatosis	28 (30.1%)	7 (6.8%)	4 (19%)
	Moderate steatosis	12 (12.9%)	35 (34%)	3 (14.3%)
	Severe steatosis	15 (16,.1%)	55 (53.4%)	13 (61.9%)

**Table 6:** Merged cluster characteristics.

Variable	Characteristic	Cluster 1	Clusters 2&3	*p*-Value
Count	-	93	124	-
Liver fibrosis	No fibrosis	71 (76.3%)	71 (57.3%)	<0.01
	F1	10 (10.8%)	7 (5.6%)	
	F2	7 (7.5%)	16 (12.9%)	
	F3	4 (4.3%)	17 (13.7%)	
	F4	1 (1.1%)	13 (10.5%)	
Liver steatosis	No steatosis	38 (40.9%)	7 (5.6%)	<0.01
	Mild steatosis	28 (30.1%)	11 (8.9%)	
	Moderate steatosis	12 (12.9%)	38 (30.6%)	
	Severe steatosis	15 (16.1%)	68 (54.8%)	

## Data Availability

The data presented in this study are available upon reasonable request from the corresponding author.

## Supplementary Materials

The following supporting information can be downloaded at: https://www.mdpi.com/article/10.3390/jcm12175657/s1, Table S1: Formulas for the scores used in our study; Table S2: Differences between genders; Table S3: General characteristics and measured parameters in patients with or without metabolic syndrome; Table S4: Differences between clusters (continuous variables); Table S5: Differences between clusters (MetS components); Figure S1: Detailed CART algorithm.
