# Supplementary material for: Hepatic Involvement across the Metabolic Syndrome Spectrum: Non-Invasive Assessment and Risk Prediction Using Machine Learning
Source: J Clin Med. 2023 Aug 30;12(17):5657. doi: 10.3390/jcm12175657 (PMC10488813; doi:10.3390/jcm12175657)
Supplement: Supplementary file 1 [file jcm-12-05657-s001.zip › jcm-2537648-supplementary.pdf]

Table S1. Formulas for the scores used in our study

Score	Formula
APRI	$100 \times \frac{\frac{AST\ (U/L)}{ULN\ AST\ (U/L)}}{PLT(10^9/L)}$
FIB4	$\frac{Age \times AST\ (U/L)}{PLT(10^9/L) \times \sqrt{ALT\ (U/L)}}$
Agile3+	$\frac{e^{-3.92368+2.29714\times\ln E(kPa)-0.00902\times PLT(10^9/L)-0.98633\times\frac{ALT\ (U/L)}{AST\ (U/L)}+1.08636\ \times[Diabetes]-0.38581\times[Gender]+0.03018\times Age\ (y)}}{1+e^{-3.92368+2.29714\times\ln E(kPa)-0.00902\times PLT\ (10^9/L)-0.98633\ \times\frac{ALT\ (U/L)}{AST\ (U/L)}+1.08636\ \times[Diabetes]-0.38581\times[Gender]+0.03018\times Age\ (y)}}$
Agile 4	$\frac{e^{7.50139+15.42498\times\frac{1}{\sqrt{E(kPa)}}-0.01378\times PLT(10^9/L)-1.41149\times\frac{ALT\ (U/L)}{AST\ (U/L)}-0.53281\times[Gender]+0.41741\times[Diabetes]}}{1+e^{7.50139+15.42498\times\frac{1}{\sqrt{E(kPa)}}-0.01378\times PLT(10^9/L)-1.41149\times\frac{ALT\ (U/L)}{AST\ (U/L)}-0.53281\times[Gender]+0.41741\times[Diabetes]}}$
HSI	$8 \times \frac{ALT(U/L)}{AST(U/L)} + BMI + 2(if\ type\ 2\ diabetes) + 2(if\ female)$

where ULN AST is the upper limit of the normal range for AST, PLT is platelet count, diabetes is 1 for patients with diabetes and 0 otherwise, and gender is 1 for males and 0 for females.

Table S2. Differences between genders

Variable	Descriptive Parameter	Gender		p-Value
		Female	Male	
BMI (kg/m²)	Mean	29.93	28.32	0.024
	StdDev	5.67	4.9	
	IQR	7.2	5.7	
	MIN	18.7	19.5	
	MAX	47.7	49.3	
	95%CI	28.93–30.93	27.3–29.34	
ALT (U/L)	Mean	33.67	37.15	P = 0.01
	StdDev	31.53	24.71	
	IQR	18	24	
	MIN	7	7	
	MAX	189	146	
	95%CI	28.12–39.23	32.01–42.3	
Platelets (10^9/L)	Mean	273.37	244.25	P < 0.01
	StdDev	78.74	69.38	
	IQR	103	83	
	MIN	53	72	
	MAX	546	441	
	95%CI	259.48–287.25	229.8–258.7	
APRI	Mean	0.36	0.41	< 0.01
	StdDev	0.39	0.33	
	IQR	0.21	0.25	
	MIN	0.08	0.07	
	MAX	3.16	2.06	
	95%CI	0.3–0.43	0.34–0.48	
	Mean	1	0.92	0.023
	StdDev	0.38	0.44	

AAR	IQR	0.46	0.43	
	MIN	0.22	0.38	
	MAX	2.38	2.38	
	95%CI	0.94–1.08	0.84–1.02	
HSI	Mean	42.04	39.2932	< 0.01
	StdDev	7.03	7.2	
	IQR	10.21	10.18	
	MIN	26.7	27.39	
	MAX	69.41	65.73	
	95%CI	40.8–43.28	37.79–40.79	

Table S3. General characteristics and measured parameters in patients with or without metabolic syndrome

Variable	Descriptive Parameter	Metabolic Syndrome		p-Value
		No	Yes	
Age (years)	Mean	53.10	62.78	< 0.01
	StdDev	14.08	10.53	
	IQR	21	12	
	MIN	19	30	
	MAX	84	85	
	95%CI	50.42–55.79	60.78–64.78	
BMI (kg/m²)	Mean	26.51	31.97	< 0.01
	StdDev	3.95	5.29	
	IQR	4.4	7	
	MIN	18.70	20.50	
	MAX	40.60	49.30	
	95%CI	25.76–27.27	30.96–32.97	
AST (U/L)	Mean	25.72	32.94	< 0.01
	StdDev	14.88	25.87	
	IQR	12	18	
	MIN	11	10	
	MAX	100	217	
	95%CI	22.88–28.56	28.03–37.86	
ALT (U/L)	Mean	30.54	39.69	< 0.01
	StdDev	23.88	35.53	
	IQR	17	25	
	MIN	1	8	
	MAX	189	183	
	95%CI	25.98–35.09	33.51–45.86	
Platelets (10^9/L)	Mean	271.21	251.19	P = 0.034
	StdDev	64.80	85.08	
	IQR	83	117	
	MIN	116	53	
	MAX	546	480	
	95%CI	258.85–283.57	235.04–267.35	
CAP (dB/m)	Mean	259.84	301.44	< 0.01
	StdDev	54.03	46.66	
	IQR	80	60	
	MIN	101	153	
	MAX	390	400	
	95%CI	249.54–270.15	292.58–310.30	
E (kPa)	Mean	5.15	8.67	< 0.01

	StdDev	3.05	5.84	
	IQR	1.6	4.9	
	MIN	2.1	2.1	
	MAX	28.6	38	
	95%CI	4.57–5.73	7.57–9.78	
APRI	Mean	0.30	0.47	< 0.01
	StdDev	0.22	0.45	
	IQR	0.16	0.31	
	MIN	0.09	0.07	
	MAX	1.60	3.16	
	95%CI	0.26–0.34	0.38–0.55	
FIB4	Mean	1.04	1.67	< 0.01
	StdDev	0.64	1.62	
	IQR	0.55	0.94	
	MIN	0.22	0.33	
	MAX	4.40	11.49	
	95%CI	0.92–1.16	1.36–1.98	
AAR	Mean	0.99	0.96	P > 0.05
	StdDev	0.42	0.39	
	IQR	0.48	0.48	
	MIN	0.44	0.22	
	MAX	2.57	2.58	
	95%CI	0.92–1.07	0.88–1.03	
Agile3+	Mean	0.1504	0.4359	< 0.01
	StdDev	0.1749	0.3010	
	IQR	0.1597	0.5468	
	MIN	0.0036	0.0101	
	MAX	0.9658	0.9977	
	95%CI	0.117–0.1838	0.3789–0.4931	
Agile4	Mean	0.0243	0.1158	< 0.01
	StdDev	0.0892	0.2228	
	IQR	0.0134	0.0878	
	MIN	0.0001	0.0003	
	MAX	0.6921	0.9610	
	95%CI	0.0073–0.6921	0.0735–0.1581	
HSI	Mean	37.33	44.41	< 0.01
	StdDev	5.69	6.84	
	IQR	8.52	9.53	
	MIN	26.7	31.30	
	MAX	51.3	69.41	
	95%CI	36.25–38.42	43.11–45.71	

Table S4. Differences between clusters (continuous variables)

Variable	Descriptive Parameter	Cluster 1	Cluster 2	Cluster 3	p-Value
Age (years)	Mean	53.62	60.5	64.76	< 0.01
	StdDev	15.11	10.94	9.15	
	IQR	23	15	15	
	MIN	19	35	35	
	MAX	84	85	85	
	95%CI	50.51–56.74	58.36–62.63	60.6–68.93	
BMI (kg/m^2)	Mean	27.61	30.3	31.43	< 0.01

	StdDev	4.95	5.12	6.76	
	IQR	6.4	6.1	11.7	
	MIN	20.3	18.7	23.9	
	MAX	47.7	46.5	49.3	
	95%CI	26.59–28.63	29.3–31.3	28.36–34.51	
AST (U/L)	Mean	24.13	33.36	32.81	< 0.01
	StdDev	11.45	27.29	17.79	
	IQR	11	18	20	
	MIN	10	12	11	
	MAX	62	217	76	
	95%CI	21.77–26.49	28.03–38.69	24.71–40.91	
ALT (U/L)	Mean	28.94	40.7	35.29	< 0.01
	StdDev	20.09	34.84	24.6	
	IQR	16	25	26	
	MIN	7	7	7	
	MAX	94	189	116	
	95%CI	24.8–33.07	33.89–47.51	24.08–46.49	
Platelets (10^9/L)	Mean	261.34	268.08	226.38	< 0.01
	StdDev	63.06	81.14	96.24	
	IQR	80	108	170	
	MIN	72	53	99	
	MAX	441	546	408	
	95%CI	248.36–274.33	252.22–283.93	182.57–270.19	
APRI	Mean	0.29	0.43	0.56	< 0.01
	StdDev	0.17	0.44	0.49	
	IQR	0.17	0.21	0.51	
	MIN	0.07	0.08	0.09	
	MAX	0.86	3.16	1.86	
	95%CI	0.26–0.33	0.34–0.51	0.34–0.79	
FIB4	Mean	1.09	1.45	2.1	< 0.01
	StdDev	0.68	1.52	1.58	
	IQR	0.74	0.78	2.23	
	MIN	0.22	0.34	0.71	
	MAX	3.71	11.49	5.86	
	95%CI	0.95–1.23	1.15–1.75	1.38–2.81	
AAR	Mean	1	0.94	1.06	0.240
	StdDev	0.42	0.4	0.4	
	IQR	0.51	0.45	0.61	
	MIN	0.45	0.22	0.38	
	MAX	2.43	2.58	1.84	
	95%CI	0.91–1.08	0.86–1.02	0.88–1.24	
HSI	Mean	38.67	42.51	42.69	< 0.01
	StdDev	6.47	7.07	8.63	
	IQR	9.83	8.53	10.37	
	MIN	26.8	26.7	30.19	
	MAX	56.74	69.41	61.80	
	95%CI	37.34–40	41.14–43.9	38.76–46.62	

Table S5. Differences between clusters (MetS components)

Variable	Descriptive Parameter	Cluster			p-Value
		1	2	3	
MetS	Frequency (%)	28 (30.1%)	63 (61,2%)	18 (85,7%)	< 0.01
WC	Frequency (%)	27 (29%)	64 (62.1%)	12 (57.1%)	< 0.01
BP	Frequency (%)	40 (43%)	67 (65%)	19 (90,5%)	< 0.01
FG/T2DM	Frequency (%)	46 (49,5%)	79 (76,7%)	17 (81%)	< 0.01
TGL	Frequency (%)	24 (25,8%)	42 (40,7%)	14 (66.7%)	< 0.01
HDL	Frequency (%)	19 (20,4%)	43 (41,7%)	13 (61.9%)	< 0.01

where WC is waist circumference ≥ 94/≥ 80 cm for men/women; BP is arterial pressure ≥ 130/85 mm Hg or treated for hypertension; FG/T2DM is fasting glucose ≥ 100 mg/dl or treated for T2DM; TGL is serum triglycerides >150 mg/dl or treated for dyslipidemia; and HDL is HDL cholesterol < 40/50 mg/dl for men/women or treated for dyslipidemia. Percentages are expressed within clusters.
