# Supplementary figures and images for: Hepatic Involvement across the Metabolic Syndrome Spectrum: Non-Invasive Assessment and Risk Prediction Using Machine Learning
Source: J Clin Med. 2023 Aug 30;12(17):5657. doi: 10.3390/jcm12175657 (PMC10488813; doi:10.3390/jcm12175657)

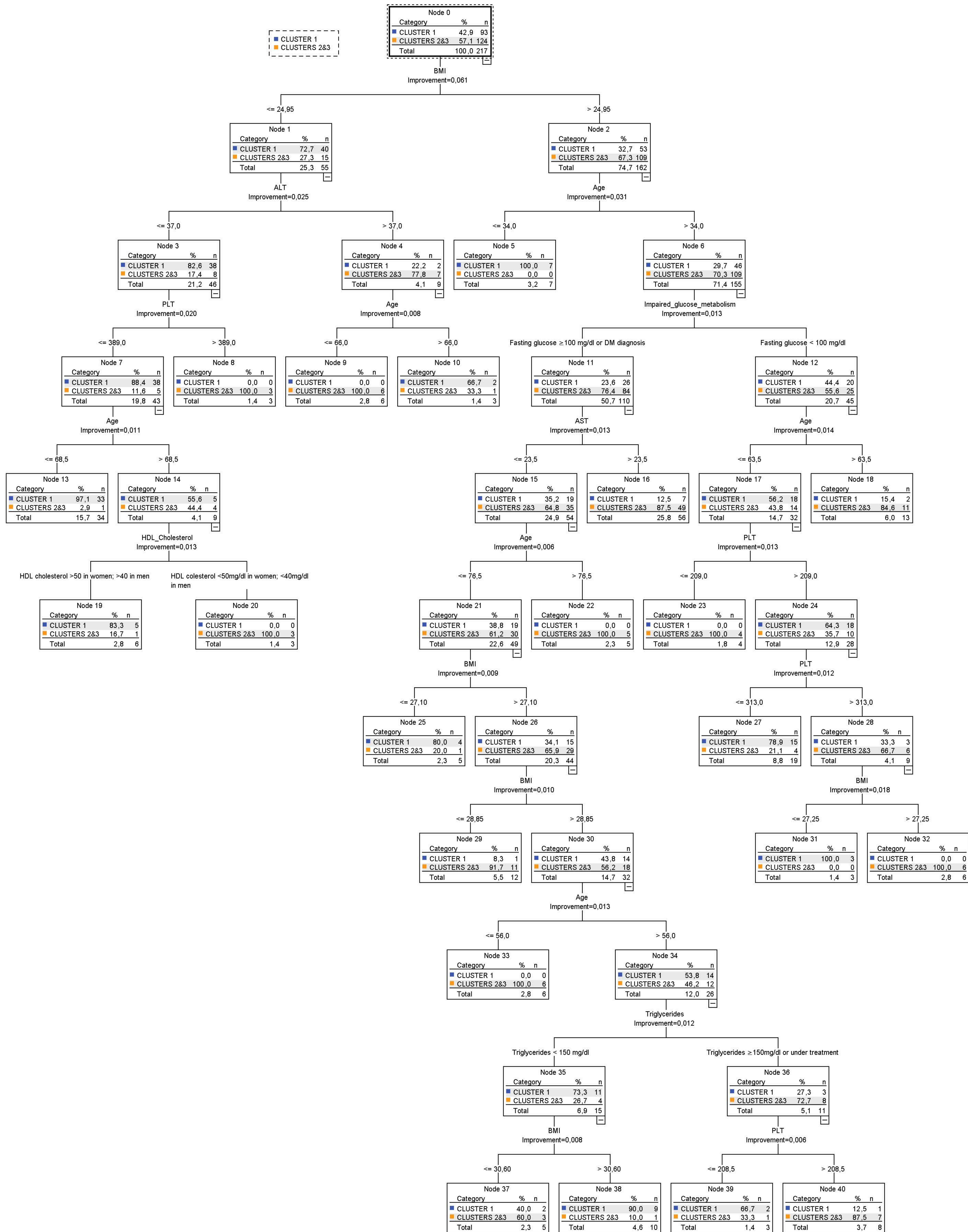

Supplement: Supplementary file 1 [file jcm-12-05657-s001.zip › Figure S1.Detailed CART algorithm.pdf]
